# Establishment and evaluation of novel prognostic biomarkers based on systemic coagulation-inflammation index and related genes in breast cancer

**DOI:** 10.3389/fimmu.2026.1836706

**Published:** 2026-07-22

**Authors:** Fucheng Li, Tian Gao, Jingqi Xia, Jianan Wang, Qiuyue Su, Yurong Chen, Yuling Ba, Siyuan Jia, Zhaoting Li, Yilin Wang, Min Xiao

**Affiliations:** 1 Department of Breast Surgery, Harbin Medical University Cancer Hospital, Harbin, Heilongjiang, China; 2 Department of Gynecological Radiotherapy, Harbin Medical University Cancer Hospital, Harbin, Heilongjiang, China

**Keywords:** breast cancer, coagulation, inflammation, intercellular communication, prognostic biomarker, systemic coagulation-inflammation index

## Abstract

**Background:**

Coagulation and inflammation play crucial roles in the initiation and progression of cancer, and they exhibit a synergistic effect. However, a hematological biomarker and risk model based on coagulation and inflammatory have not yet been established in breast cancer.

**Methods:**

This study retrospectively analyzed 749 breast cancer patients at our institution. We established a coagulation-inflammation-related genes (CIRGs) risk model using the TCGA-BRCA dataset as the training set and GEO datasets (GSE20685, GSE21653) as the testing sets. Immunohistochemical staining was performed on tumor tissues to validate protein-level expression of key genes. Additionally, single-cell RNA sequencing (scRNA-seq) was applied to investigate the expression of the CIRGs and explore intercellular communication differences.

**Results:**

All enrolled cases were stratified by median systemic coagulation-inflammation index (SCI) into high- and low-SCI groups. The low-SCI group had longer disease-free survival and overall survival than the high-SCI group, with higher SCI levels noted in triple-negative breast cancer (TNBC). SCI exhibited a linear correlation with survival outcomes and superior survival predictive performance compared to the platelet-to-lymphocyte ratio. LASSO regression selected 21 prognosis-related CIRGs to construct a prognostic model, which showed robust predictive efficacy across three datasets. Tumor microenvironment analysis indicated that the high-risk group was associated with immune suppression. Drug sensitivity analysis identified 6 potential candidate drugs for low- and high-risk groups. Four hub genes (*ABCA1*, *IL1R1*, *SERPINE1*, and *HPN*) were identified among CIRGs, showing strong correlations with tumor stage and prognosis. scRNA-seq analysis revealed high expression of the CIRGs in myeloid, endothelial, and epithelial cells, with higher AUCell scores of the CIRGs in TNBC. Significant intercellular communication differences were observed between the two risk groups, especially in fibroblast/endothelial cell-to-T/B cell signaling pathways.

**Conclusion:**

This study deeply explores the roles and clinical significance of coagulation and inflammation in breast cancer. The hematological indicator SCI and the CIRGs risk model can serve as reliable biomarkers for breast cancer personalized treatment.

## Introduction

Breast cancer is the most prevalent malignant tumor in women globally and the leading cause of cancer-related mortality (1). Despite remarkable advances in therapeutic strategies including surgery, neoadjuvant chemotherapy (NACT), immunotherapy, and targeted therapy (2), the inherent heterogeneity of breast cancer results in substantial prognostic variability among patients (3). Currently, pathological parameters routinely used in clinical practice—including tumor staging and hormone receptor status, are widely applied for prognostic evaluation, yet they cannot fully capture the complex biological mechanisms underlying tumor progression, leading to suboptimal predictive performance. Accordingly, the identification of robust prognostic biomarkers is of paramount clinical importance.

A growing body of evidence indicates that coagulation and inflammation exert pivotal roles in tumor initiation and progression (4–7). Coagulation dysfunction, characterized by enhanced platelet activation and fibrin accumulation, can create a tumor-promoting microenvironment that drives cancer cell proliferation, invasion, and metastasis (8, 9). Likewise, inflammatory responses mediated by cytokines, chemokines, and immune cells facilitate tumor angiogenesis and immune evasion, thereby promoting tumor progression (10). Our group previously demonstrated that the pan-immune-inflammatory value, a systemic hematological inflammatory indicator, is predictive of survival outcomes in young breast cancer patients (11). Notably, extensive crosstalk exists between the coagulation and inflammatory systems: activation of the coagulation system favors the initiation of inflammatory responses, establishing a synergistic loop that amplifies cancer progression (12, 13). Clinical evidence from melanoma studies demonstrates that anticoagulants augment the therapeutic efficacy of immune checkpoint blockade (ICB) (14). Despite confirmed associations between coagulation and inflammation, there remains a lack of reliable hematological prognostic markers integrating the coagulation-inflammation system, as well as robust prognostic models based on coagulation-inflammation-related genes (CIRGs)—a critical gap that impedes the advancement of personalized therapeutic strategies.

As one of the few hematological biomarkers that capture both coagulation and inflammatory status, the platelet-to-lymphocyte ratio (PLR) exhibits inconsistent prognostic value across different cancer cohorts, limiting its reliability in clinical application (15, 16). The systemic coagulation-inflammation index (SCI) is a novel hematological indicator that can accurately reflect coagulation and inflammation dynamics in patients with chronic obstructive pulmonary disease and coronary artery disease (17, 18), thereby enabling prognostic assessment. However, the prognostic relevance of SCI in cancer patients remains unexplored to date. In a study focusing on hepatocellular carcinoma, 11 coagulation-related genes (CRGs) were selected to establish a risk score; this score was found to be significantly correlated with the efficacy of immunotherapy and capable of predicting prognosis (19). In colorectal carcinoma, a separate study established a prognostic model using 11 inflammation-related genes (IRGs), which showed robust capacity to stratify patients by prognostic risk (20). While limited studies have explored the prognostic roles of coagulation- or inflammation-related genes in breast cancer (21, 22), a comprehensive gene signature integrating the coagulation-inflammation for long-term survival prediction remains to be established.

In this study, we first evaluated the prognostic value of the SCI in 749 breast cancer patients treated with NACT, in comparison with PLR. Subsequently, we constructed a CIRGs risk model based on TCGA and GEO datasets, and integrated it with clinicopathological features to develop a nomogram for accurate survival prediction. We next explored the associations of this model with clinicopathological characteristics, tumor immune landscape, and drug sensitivity. We further identified four hub genes (*ABCA1*, *IL1R1*, *SERPINE1*, and *HPN*) and validated their clinical value. Finally, single-cell RNA sequencing (scRNA-seq) was employed for analyzing the expression characteristics of the CIRGs risk model across distinct cell subsets and differences in intercellular communication. Collectively, our study identifies two novel prognostic biomarkers to inform personalized therapeutic strategies for breast cancer.

## Patients and methods

### Patient selection and data collection

This clinical cohort at our institution adhered to the ethical principles of the 1975 Declaration of Helsinki, and all patients signed informed consent forms (including provisions for secondary data use) prior to treatment initiation. The study was approved by the Ethics Committee of the Harbin Medical University Cancer Hospital (Approval No. KY2024-76).

We retrospectively analyzed breast cancer patients who received NACT at our hospital between November 2011 and January 2019, and clinical data were retrieved from the hospital database. Inclusion criteria were: (I) invasive breast malignancy via core needle biopsy; (II) having completed biochemical blood tests within 1 week prior to NACT; (III) receipt of NACT and subsequent surgery at the same institution; and (IV) availability of complete follow-up, pathological, and clinical data. Exclusion criteria included: (I) inaccurate or incomplete medical records; (II) presence of distant metastases. Finally, 749 patients were involved in our research (**Table 1**).

**Table 1:** Clinicopathological characteristics of patients with different SCI groups.

Variable	SCI ≤ 106.5 (n=375)	SCI>106.5 (n=374)	*P* value
Age			0.587
<40	112 (29.9%)	124 (33.2%)	
40 to 60	220 (58.7%)	212 (56.7%)	
≥60	43 (11.5%)	38 (10.2%)	
BMI			0.235
<25	251 (66.9%)	228 (61%)	
25 to 30	105 (28%)	124 (33.2%)	
≥30	19 (5.1%)	22 (5.9%)	
cT			0.728
T1 + T2	298 (79.5%)	302 (80.7%)	
T3 + T4	77 (20.5%)	72 (19.3%)	
cN			0.123
N0	46 (12.3%)	32 (8.6%)	
N1 - N3	329 (87.7%)	342 (91.4%)	
HR			0.856
Negative	120 (32%)	123 (32.9%)	
Positive	255 (68%)	251 (67.1%)	
HER-2			0.616
Negative	265 (70.7%)	257 (68.7%)	
Positive	110 (29.3%)	117 (31.3%)	
Subtype			0.341
ER+	213(56.8%)	194(51.9%)	
HER2+	110(29.3%)	117(31.3%)	
TNBC	52(13.9%)	63(16.8%)	
Ki-67			0.677
≤14%	117 (31.2%)	123 (32.9%)	
>14%	258 (68.8%)	251 (67.1%)	
Surgery			0.992
Mastectomy	349 (93.1%)	347 (92.8%)	
Conservative surgery	26 (6.9%)	27 (7.2%)	
p53			0.688
Negative	226 (60.3%)	219 (58.6%)	
Positive	149 (39.7%)	155 (41.4%)	
pCR			0.547
No	290 (77.3%)	297 (79.4%)	
Yes	85 (22.7%)	77 (20.6%)	
PLR			<0.001
≤132.1	252 (67.2%)	123 (32.9%)	
>132.1	123 (32.8%)	251 (67.1%)	

From our hospital’s medical records, we retrieved diagnostic data, demographic information, pathology reports, clinical TNM stages, and laboratory test results. The laboratory tests included SCI (platelet count × fibrinogen level/white blood cell count) (17, 18) and PLR (platelet count/lymphocyte count) (15, 16), which were conducted 1 week before NACT. The absence of any residual invasive cancer cells in the breast and regional lymph nodes was referred to as pathological complete response (pCR).

Original RNA-sequencing and clinical data from TCGA_BRCA cohort (n=1,231) was downloaded from TCGA database (https://portal.gdc.cancer.gov/). The GEO database provided the gene expression microarrays for GSE20685 (n=327) and GSE21653 (n=252) (https://www.ncbi.nlm.nih.gov/geo/). Single-cell transcriptional data from GSE176078 (23) (n=26) was also collected from GEO database.

We retrieved CRGs by searching for the term “coagulation” on the GeneCards database (https://www.genecards.org/). Based on the threshold of a relevance score > 3, a total of 611 CRGs were obtained (**Supplementary Table 1**). A total of 200 IRGs were acquired from MSigDB database (https://www.gsea-msigdb.org/) (**Supplementary Table 2**).

### Follow-up

Patients from the clinical cohort at our institution were followed up every 3 months during the initial 2-year period after treatment, and the follow-up interval was adjusted to every 6 months for the subsequent 3-year period. Disease-free survival (DFS) was defined as the interval from initial diagnosis to local recurrence, distant metastasis, or all-cause death. Overall survival (OS) was defined as the interval from initial diagnosis to last follow-up or death.

### Construction and validation of the CIRGs risk model

Prognostic genes were selected using least absolute shrinkage and selection operator (LASSO) regression analysis. LASSO Cox proportional hazards regression analysis was performed using the glmnet package (version 4.1-9). To minimize the risk of overfitting and ensure model robustness, 10-fold cross-validation was employed for parameter optimization. To eliminate bias introduced by random partitioning, the entire 10-fold cross-validation procedure was independently repeated 100 times, and “deviance” was used as the metric for evaluating model performance during cross-validation. The lambda.min value was selected as the optimal regularization parameter to generate the most accurate model. In this study, the lambda.min criterion was achieved at lambda = 0.01403887, at which point 21 genes with non-zero coefficients were identified by the model, constituting the final CIRGs prognostic signature. The risk score for each patient was calculated using a weighted summation method based on the 21 selected genes and their corresponding LASSO regression coefficients. The risk score for each patient was calculated according to the following formula: *Risk score* = *Σ* (*Coefi* × *Exp*). The median risk score was then used to categorize all patients into high-risk and low-risk groups. To assess the accuracy and consistency of the prognostic model, Kaplan-Meier survival curves, receiver operating characteristic (ROC) curves, nomograms, calibration curves, and decision curve analysis (DCA) curves were generated using the R packages survival (Version 3.8-3), timeROC (Version 0.4), regplot (Version 1.1), rms (Version 8.0-0), survminer (Version 0.5.0), and ggDCA (Version 1.2).

### Immune microenvironment analysis

We initially investigated immune cell infiltration across various CIRGs subgroups by means of single-sample gene set enrichment analysis (ssGSEA). Subsequently, the CIBERSORT (24) algorithm was applied to assess immune cell infiltration through the “IOBR” R package (version 0.99.0).

### Drug sensitivity

The “oncoPredict” (25) R package was used to predict drug responses. This allowed us to identify drugs with high sensitivity across three bulk datasets.

### Construction and validation of the simplified 4-hub-gene prognostic model

A comprehensive gene-clinical prognostic signature was constructed based on the multivariate Cox proportional hazards regression model, which integrated the mRNA expression levels of the four core hub genes (*ABCA1*, *IL1R1*, *SERPINE1* and *HPN*) and three independent prognostic clinical factors (age, T stage and N stage). Risk Score = 0.1414×*ABCA1* + 0.0047×*IL1R1* + 0.0523×*SERPINE1* - 0.1209×*HPN* + 0.3444×(Age=51-65) + 1.1976×(Age>65) + 0.0262×(T stage=T2) + 0.3108×(T stage=T3) + 0.6215×(T stage=T4) + 0.8394×(N stage= Node-positive). The risk score for each patient was calculated using the above formula, where higher scores indicate a higher risk of all-cause mortality. Patients were stratified into high-risk and low-risk groups using the median risk score as the predefined cutoff value.

### Immunohistochemistry

Formalin-fixed paraffin-embedded (FFPE) tissue samples from 5 patients with early-stage breast cancer (I stage) and 5 patients with locally advanced breast cancer (II/III stage) were selected from our institutional biobank for immunohistochemical staining. Tissue sections were dewaxed in xylene and rehydrated through graded ethanol solutions, followed by quenching of endogenous peroxidase activity with 3% hydrogen peroxide. The sections were then incubated with anti-ABCA1 (1:1000, Proteintech, #26564-1-AP), anti-IL-1R1 (1:1000, Proteintech, #27348-1-AP) and anti-SERPINE1 (1:200, Proteintech, #13801-1-AP) at 4 °C for 24 hours. ImageJ software was utilized for semi-quantitative analysis.

### scRNA-seq data processing

In the scRNA-seq analysis, all samples were sequenced via the 10X Genomics scRNA-seq technology. The “Seurat” R package (version 5.3.0) in R software was used to process the scRNA-seq data. Genes and cells were filtered according to the following criteria: a gene must be expressed in at least 5 cells, and each cell must have 200 to 5000 detected genes; cells with mitochondrial gene content below 20% were retained, while the cell cycle genes were also considered. Concurrently, the “scDblFinder” (26) R package was used to remove doublets. Data normalization was performed using the LogNormalize function, and batch effects were eliminated using the Harmony R package (version 1.2.3). A total of 14 clusters were identified via the Uniform Manifold Approximation and Projection (UMAP) algorithm, and cell types were ultimately annotated based on significantly differentially expressed marker genes.

### Single-cell gene visualization and gene set activity scoring

The Nebulosa (27) R package (version 1.16.0) visualized the single-cell data based on kernel density estimation, which we used to visualize the four hub genes. The AUCell R package (version 1.28.0) was applied to score the activity of CIRGs-related gene sets.

### Cell–cell interaction analysis

To analyze cell-cell communication, we applied the CellChat (28) R package to compare intercellular interactions between different CIRGs prognostic groups.

### Statistical analysis

Differences between the low and high SCI groups were compared using the χ^2^-test. Cox proportional hazards regression modeling was used to calculate hazard ratios and 95% confidence intervals (CI). In order to investigate the size-response relationship between SCI and patient’s recurrence as well as mortality, we employed a restricted cubic spline (RCS) regression model containing four nodes. We assessed nonlinearity via the likelihood ratio test. Curves for DFS and OS were generated with the Kaplan-Meier approach and log-rank test.

The Wilcoxon test was used to compare continuous variables between two independent samples, while the Kruskal-Wallis test was employed to evaluate and compare continuous variables across three or more groups. Differences were considered statistically significant when *P* < 0.05 (*, *P*<0.05;**, *P*<0.01;***, *P*<0.001; ****, *P*<0.0001). All statistical analyses were performed using the R software (version 4.4.1).

## Results

### The baseline of breast cancer patients at our institution

**Figure 1:**
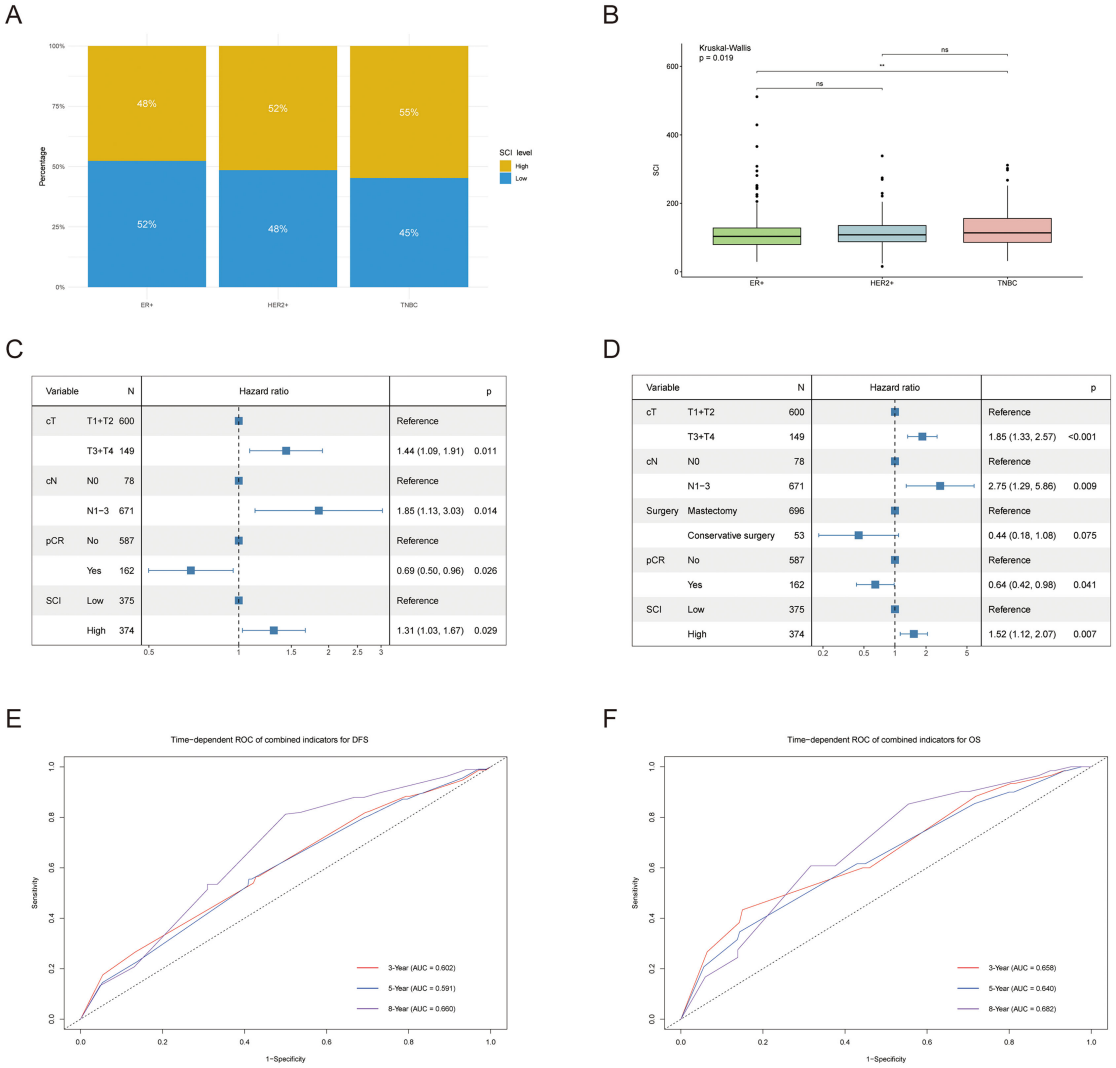
Survival analyses of the SCI for the prediction of DFS and OS. **(A)** Stacked bar chart showing the proportional distribution of SCI levels across different breast cancer subtypes. **(B)** Box plot showing the distribution of SCI values across different subtypes. **(C)** Multivariate cox regression survival analyses of the SCI for the prediction of DFS. **(D)** Multivariate cox regression survival analyses of the SCI for the prediction of OS. **(E)** Time-dependent ROC curves of combined indicators for DFS. **(F)** Time-dependent ROC curves of combined indicators for OS. Kruskal–Wallis tests was used to assess the significance of differences in panel **(B)** **P < 0.01.

We enrolled 749 consecutive patients with breast cancer treated with NACT, with a median baseline SCI of 106.5 at diagnosis. Patients were stratified into high- and low-SCI groups using the median SCI as the cut-off value. Comparable baseline characteristics were observed between the two groups, including similar age, body mass index (BMI), cT stage, cN stage, hormone receptor (HR), human epidermal growth factor receptor-2 (HER-2), molecular subtype, Ki-67, p53, surgery, and pCR statuses. No significant correlation was identified between SCI and clinicopathological characteristics in this study. However, SCI was strongly correlated with PLR (*P* < 0.001), reflecting the consistency between the two indices in assessing the body’s inflammatory and coagulation function levels. The baseline characteristics of the patients are summarized in **Table 1**. Notably, when analyzed as a continuous variable, SCI levels were higher in triple-negative breast cancer (TNBC) patients than in estrogen receptor (ER) positive patients (**Figures 1A, B**). This finding indicates that patients with TNBC may exhibit a more pronounced systemic coagulation and inflammatory state.

### Survival analysis of patients at our institution

The median follow-up duration was 68 months, ranging from 3 to 141 months. By the end of follow-up, the mean values of DFS and OS in 749 patients were 64.02 and 72.23 months. The univariate survival analysis demonstrated that cT stage, cN stage, surgery, pCR, and SCI were significantly associated with survival (*P* < 0.05, **Supplementary Table 3**). However, the multivariate Cox proportional hazard models survival analysis demonstrated that only cT stage (*P* = 0.011; **Figure 1C**; *P* < 0.001; **Figure 1D**), cN stage (*P* = 0.014; **Figure 1C**; *P* = 0.009; **Figure 1D**), pCR (*P* = 0.026; **Figure 1C**; *P* = 0.041; **Figure 1D**), and SCI (*P* = 0.029; **Figure 1C**; *P* = 0.007; **Figure 1D**) were independent predictors for DFS and OS.

The predictive ability of the combined indicators for DFS and OS at different durations (3 years, 5 years, and 8 years) was demonstrated through time-dependent ROC curves. The comprehensive indicators, including the SCI, showed good predictive ability for 8-year DFS and OS (area under the curve (AUC) = 0.660, **Figure 1E**; AUC = 0.682; **Figure 1F**).

**Figure 2:**
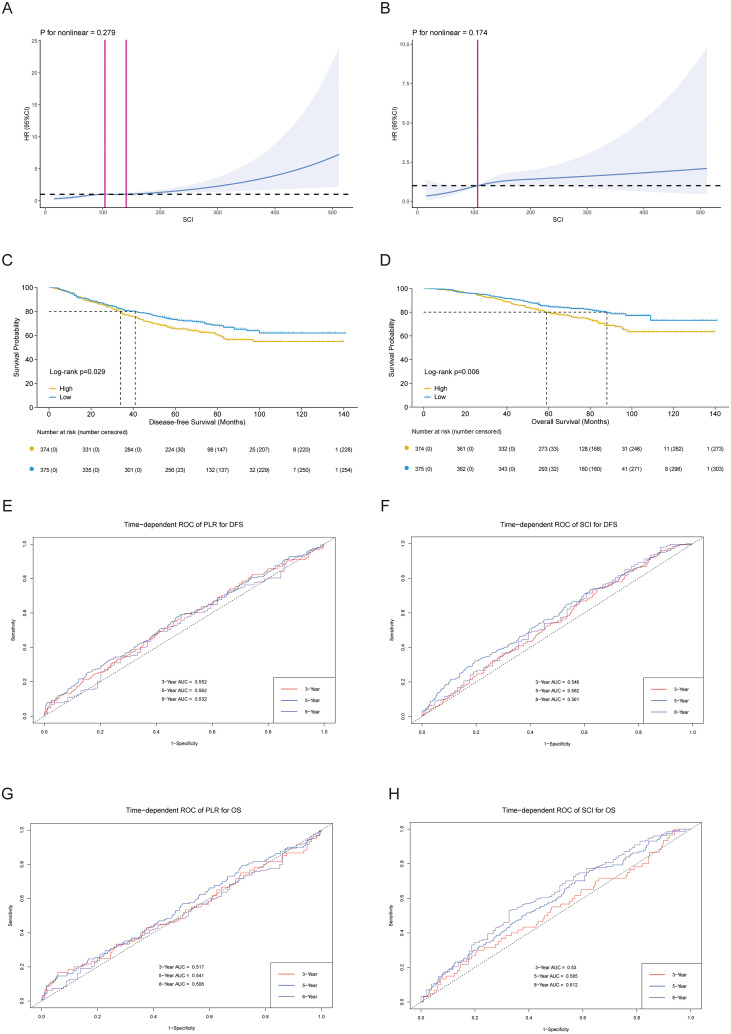
The relationship between SCI and DFS and OS. **(A)** RCS models representing the associations between SCI and DFS. **(B)** RCS models representing the associations between SCI and OS. **(C)** Kaplan-Meier analysis of the relationship between SCI and DFS. **(D)** Kaplan-Meier analysis of the relationship between SCI and OS. **(E)** Time-dependent ROC curves of PLR for DFS. **(F)** Time-dependent ROC curves of SCI for DFS. **(G)** Time-dependent ROC curves of PLR for OS. **(H)** Time-dependent ROC curves of SCI for OS.

We further employed the RCS model to assess the patterns of dose-response pattern between SCI and survival endpoints. After adjustment for cT stage, cN stage, and pCR status, the dose-response correlations between SCI and survival outcomes exhibited a consistent pattern. No significant nonlinear correlation was observed between SCI levels and either survival endpoint (**Figures 2A, B**). Specifically, elevated SCI exhibited a significant linear association with both DFS (*P* for nonlinearity = 0.279; **Figure 2A**) and OS (*P* for nonlinearity = 0.174; **Figure 2B**). The hazard ratio for DFS and OS exhibited a continuous linear upward trend with increasing SCI levels. When SCI was below 103.9, the hazard ratio for DFS was less than 1, indicating that patients with low SCI may have a lower recurrence risk than the reference level; in contrast, when SCI exceeded 141, the hazard ratio for DFS was greater than 1. For OS, the hazard ratio was less than 1 when SCI was below 106.55, suggesting that patients with low SCI may have a lower mortality risk than the reference level; conversely, the hazard ratio for OS was greater than 1 when SCI exceeded 106.55. Notably, when SCI was below 103.9 and 106.55 respectively, the 95% confidence intervals of the hazard ratios for DFS and OS both included the reference value of 1, indicating no statistically significant difference in survival risk compared with the reference level.

Kaplan-Meier survival analysis with the log-rank test demonstrated that DFS and OS were significantly shorter in breast cancer patients with high SCI (*P* = 0.029; **Figure 2C**; *P* = 0.006; **Figure 2D**). Notably, we found that SCI outperformed PLR as a prognostic indicator for DFS and OS, particularly for long-term survival (**Figures 2E–H**). To accurately evaluate the predictive efficacy of these two coagulation-inflammation-related hematological biomarkers, we employed the DeLong test to conduct comparisons of AUC values. Significant differences in AUC values between SCI and PLR were observed for both 5-year and 8-year OS (Z = 2.077, *P* = 0.038; Z = 5.620, *P* < 0.001; **Table 2**). However, neither SCI nor PLR was significantly associated with pCR, indicating that SCI does not reliably predict the therapeutic efficacy of NACT. (*P* = 0.490; *P* = 0.296; **Supplementary Table 4**).

**Table 2:** Comparison of prognostic ability of SCI and PLR (SCI vs. PLR).

Time	Difference of AUC	Z statistic	*P* value
3-Year (DFS)	-0.006	-0.261	0.794
5-Year (DFS)	0.020	0.963	0.336
8-Year (DFS)	0.029	1.509	0.131
3-Year (OS)	0.013	0.631	0.528
5-Year (OS)	0.044	2.077	**0.038**
8-Year (OS)	0.106	5.620	**<0.001**

Bold values indicate statistical significance at P < 0.05.

### Construction and validation of the coagulation- inflammation related prognostic risk model

**Figure 3:**
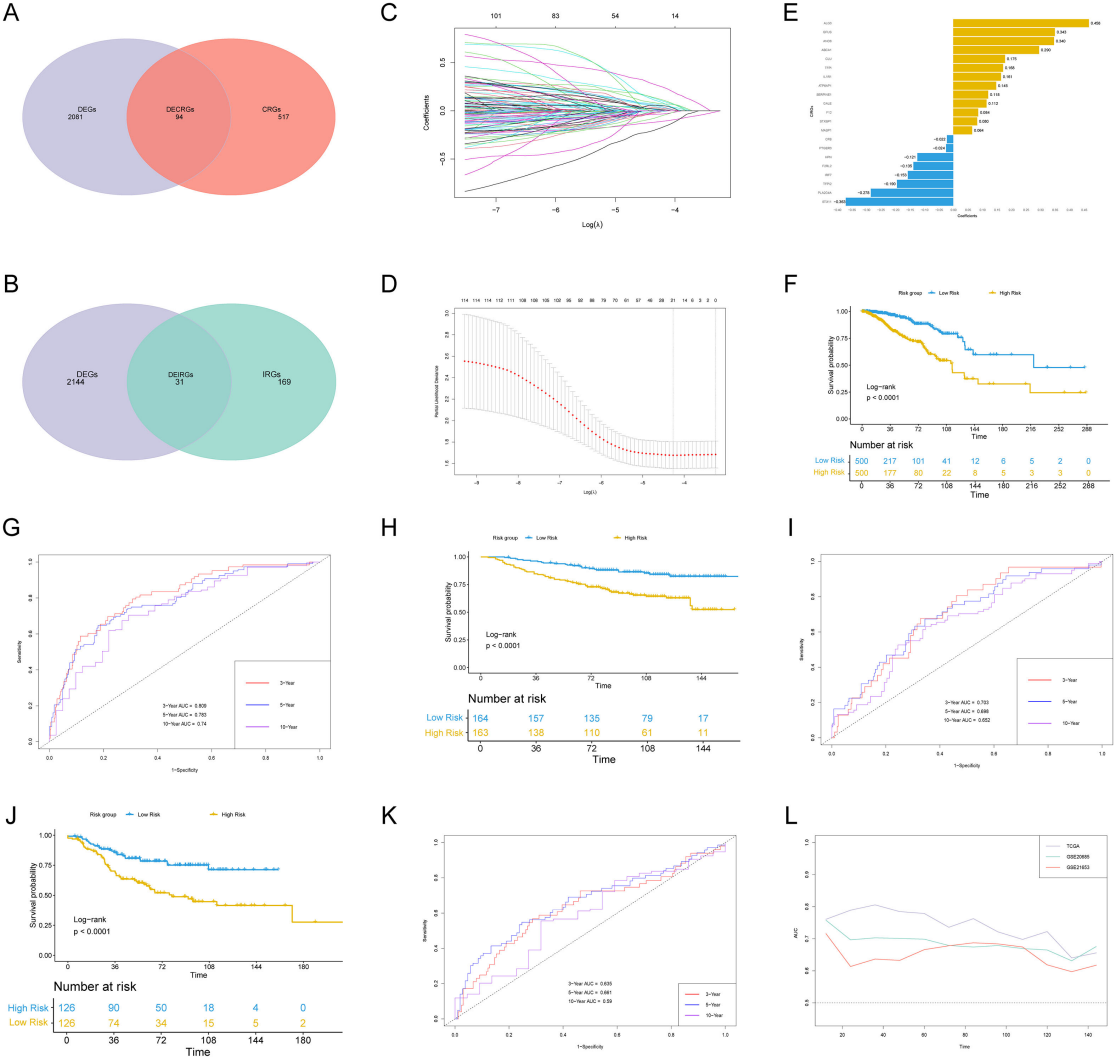
Construct a risk model for CIRGs and validate its prognostic value. **(A)** Venn plot showing the overlap of DECRGs. **(B)** Venn plot showing the overlap of DEIRGs. **(C, D)** The LASSO Cox regression of DECIRGs. **(E)** 21 CIRGs with their regression coefficients. Kaplan-Meier survival curves of the 21 prognostic CIRGs in the **(F)** TCGA cohort, **(H)** GSE20685 dataset, and **(J)** GSE21653 dataset. Time-dependent ROC curves for the risk model for 3-, 5-, and 10-year OS and the corresponding AUC values in the **(G)** TCGA cohort, **(I)** GSE20685 dataset, and **(K)** GSE21653 dataset. **(L)** Line chart showing the fluctuations in the AUC over time across the three datasets.

After excluding samples with duplicate patients and those lacking relevant clinical data from the TCGA-BRCA dataset, differential expression analysis was performed on 1,000 tumor specimens and 61 paired adjacent normal tissues, leading to the identification of a total of 2,175 differentially expressed genes (DEGs) (**Figures 3A, B**). These DEGs were intersected with CRGs and IRGs, respectively, resulting in 94 differentially expressed coagulation-related genes (DECRGs) (**Figure 3A**) and 31 differentially expressed inflammation-related genes (DEIRGs) (**Figure 3B**). The union of DECRGs and DEIRGs, termed differentially expressed coagulation-inflammation related genes (DECIRGs), consisting of 114 genes in total, was used as the gene set for subsequent model construction.

As shown in **Figures 3C, D**, we conducted LASSO Cox regression analysis on DECIRG-related genes and identified 21 genes for establishing the risk model; these genes were formally defined as CIRGs. The regression coefficients of the 21 CIRGs are presented in **Figure 3E**; **Supplementary Table 5**. The risk signature score was calculated as a weighted sum of each gene’s expression level multiplied by its corresponding regression coefficient. Of the 21 CIRGs, 13 exhibited positive associations with the risk score, while the remaining 8 showed negative associations; they were designated as CIRG-positive and CIRG-negative genes, respectively. Kaplan-Meier survival analysis demonstrated that patients in the high-risk score group had significantly poorer survival outcomes than those in the low-risk group (*P* < 0.0001; **Figure 3F**). Additionally, ROC curves were used to assess the predictive performance of the risk model. The AUC values of the risk model were 0.809 for 3-year survival, 0.783 for 5-year survival, and 0.74 for 10-year survival (**Figure 3G**). These findings indicate that the CIRGs-based risk model exhibits favorable predictive performance, particularly for short-term survival.

Subsequently, we validated the predictive performance of the CIRGs-based model in two independent external cohorts. Kaplan-Meier survival analysis demonstrated that patients in the high-risk group had significantly increased mortality risk (*P* < 0.001; **Figures 3H, J**). In the GSE20685 cohort, the CIRGs-based model achieved AUC values of 0.703 for 3-year survival, 0.698 for 5-year survival, and 0.652 for 10-year survival (**Figure 3I**). In the GSE21653 cohort, the corresponding AUC values of the model were 0.635 at 3 years, 0.661 at 5 years, and 0.590 at 10 years (**Figure 3K**). Results from the TCGA cohort and the two external validation cohorts were consistent, indicating that our CIRGs-based model has considerable stability in predicting the survival of breast cancer patients. As illustrated in **Figure 3L**, we evaluated the temporal trend of the model’s predictive performance across the three cohorts. Overall, the performance of different datasets exhibited stability patterns with notable differences: the GSE20685 cohort showed the most stable performance, with its AUC values fluctuating slightly around 0.70 without significant upward or downward trends, indicative of robust predictive capacity. This finding underscores the critical importance of validating prognostic models in multicenter, heterogeneous cohorts to support clinical translation.

### Development and validation of nomogram-based model

In addition to survival outcomes, we generated a heatmap to visualize the distribution of clinicopathological features between the two risk groups (**Supplementary Figure 1A**). The heatmap revealed significant between-group differences in survival status and tumor stage, whereas no significant difference in age was detected. Box plots showed that the risk scores exhibited good separation across TNM stages (**Supplementary Figure 1B**). We constructed a nomogram in the TCGA cohort by integrating clinicopathological variables and risk scores, to enable precise quantification of 3−, 5−, and 10−year OS probabilities (**Supplementary Figure 1C**). This nomogram enables accurate risk stratification for high-risk patients and may inform clinical treatment decision-making. The nomogram exhibited excellent discriminative and calibration abilities in both the GSE20685 and GSE21653 validation sets (**Supplementary Figures 1F, I**). Notably, the risk score consistently served as a robust predictive factor. Calibration plots based on 1000 bootstrap resamples across the three cohorts showed minimal deviation from the 45° reference line at 3, 5, and 10 years, indicating reliable model performance (**Supplementary Figures 1D, G, J**). DCA verified that the nomogram yielded the highest net benefit in predicting 5-year OS probabilities and outperformed other clinical indicators in effectiveness (**Supplementary Figure 1E, H, K**). These findings illustrate that the nomogram has reliable prognostic value and can serve as a practical tool to support clinical decision-making.

### Tumor immune microenvironment of the CIRGs risk model

**Figure 4:**
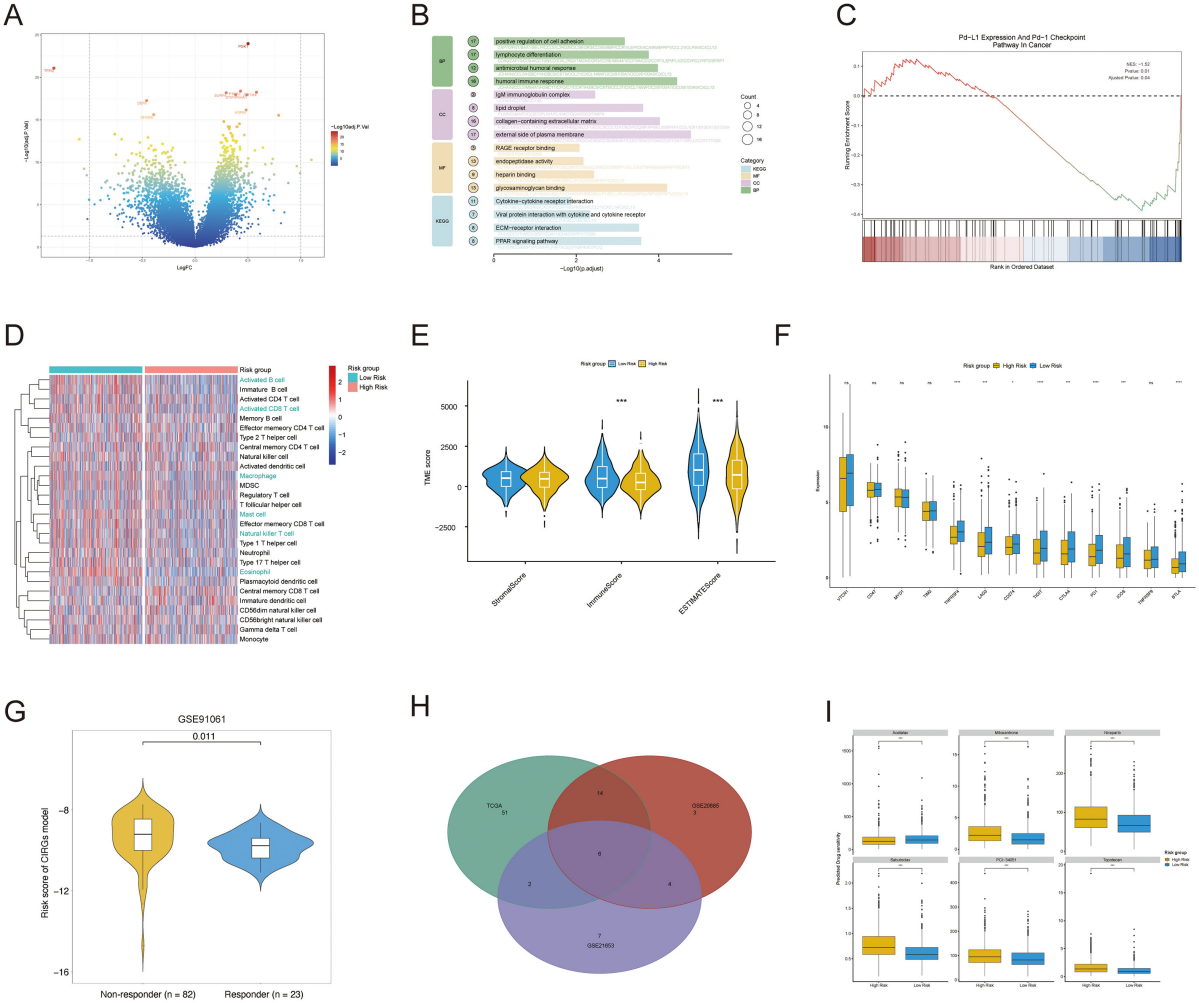
Immune landscape and drug sensitivity analysis of different risk groups. **(A)** Volcano plot showing DEGs between the high and low risk group. **(B)** Enrichment analysis of DEGs in biological process (BP), cellular component (CC), molecular function (MF), and KEGG pathways. **(C)** GSEA plot showing the enrichment of the PD-L1 expression and PD-1 checkpoint pathway. **(D)** Heatmap showing the infiltration of 28 infiltrating immune cells in two risk groups. **(E)** Violin plots showing the different distributions of stromal score, immune score and ESTIMATE score. **(F)** Box plot showing the expression differences of 13 immune checkpoint molecules between the high-risk group and the low-risk group. **(G)** Violin plots with embedded boxplots for comparing CIRGs model risk scores between treatment non-responders and responders in the GSE91061 dataset. **(H)** Venn diagram showing the overlap of 6 sensitive drugs with significant therapeutic effects across the 3 datasets. **(I)** Drug sensitivity analysis of 6 sensitive drugs in different risk groups in the TCGA database. Wilcoxon rank-sum test was used to assess the significance of differences in panels **E**, **F**, **G**, and **I**. ****P < 0.0001, ***P < 0.001, **P < 0.01, *P < 0.05.

Through comparative analysis of gene expression profiles between high- and low-risk groups, we identified 174 DEGs. The top 10 genes with significant differences were labeled in the volcano plot (**Figure 4A**). Among these DEGs, PGK1 was highly expressed in the high-risk group, which was associated with poor prognosis, chemoresistance, and tumor progression (29). TFPI2 showed high expression in the low-risk subgroup; this gene typically acts as a tumor suppressor and serves as an important inhibitor of tumor metastasis (30). GO analysis revealed that the biological processes, cellular components, and molecular functions of the DEGs primarily included positive regulation of cell adhesion, lymphocyte differentiation, external side of plasma membrane, endopeptidase activity, and glycosaminoglycan binding (**Figure 4B**). KEGG analysis indicated that the pathway significantly enriched in DEGs was cytokine-cytokine receptor interaction (**Figure 4B**). GSEA revealed that all top 10 enriched pathways were overrepresented in the low-risk subgroup, predominantly comprising immune response-related pathways such as immune receptor activity, cell killing, and adaptive immune response (**Supplementary Figure 2A**). Further GSEA confirmed that the low-risk subgroup was significantly associated with the anti-PD-1 therapeutic pathway (*P* = 0.04; **Figure 4C**).

To investigate the association between the CIRGs-related prognostic risk model and the landscape of immune, we evaluated immune cell infiltration in the TCGA cohort using the ssGSEA and CIBERSORT algorithms, and analyzed the discrepancies between the two risk groups. Our results showed that the low-risk group exhibited higher levels of activated B cells, activated CD8+ T cells, macrophages, mast cells, natural killer cells, and eosinophils compared with those in the high-risk group (**Figure 4D**). In contrast, the high-risk group had a higher infiltration level of M2 macrophages (**Supplementary Figure 2B**), which have been shown to be associated with poor prognosis in cancer patients (31). Concurrently, the low-risk group demonstrated significantly higher immune score and estimation of stromal and immune cells in malignant tumors using expression data (ESTIMATE) score (*P* < 0.001), showing elevated immunogenicity and immune activity within the tumor microenvironment (TME) of the TCGA cohort (**Figure 4E**). Given that immune checkpoint molecules serve as robust predictors of tumor immunotherapeutic response (32), we further analyzed differential expression of these molecules between the two populations. In the low-risk population, our data revealed that 8 out of 13 immune checkpoint molecules were upregulated (**Figure 4F**). In the GSE91061 cohort (33), risk scores derived from the CIRGs model were significantly higher in non-responders than in responders, suggesting that this model may serve as a candidate predictor for immunotherapy benefit (**Figure 4G**). Collectively, these observations suggest that patients may be more likely to benefit from immunotherapeutic strategies targeting immune cells and immune checkpoint molecules in the low-risk group.

### Drug sensitivity analysis in different CIRGs risk groups

To facilitate the clinical treatment guidance for the CIRGs risk model, we performed drug sensitivity analysis. We identified 73 highly sensitive drugs in the TCGA cohort, 27 in the GSE20685 cohort, and 19 in the GSE21653 cohort (*P* < 0.001). Subsequently, Venn diagram analysis was conducted to identify potential candidate drugs shared across the three datasets, yielding 6 drugs (**Figure 4H**). Furthermore, patients with higher CIRGs risk scores exhibited greater sensitivity to mitoxantrone, niraparib, sabutoclax, PCI-34051, and topotecan, whereas those with lower CIRGs risk scores showed higher sensitivity to acetalax (**Figure 4I**). In summary, these findings provide novel insights for developing personalized treatments for patients in different risk groups.

### Construction and validation of the simplified 4-hub-gene prognostic model

**Figure 5:**
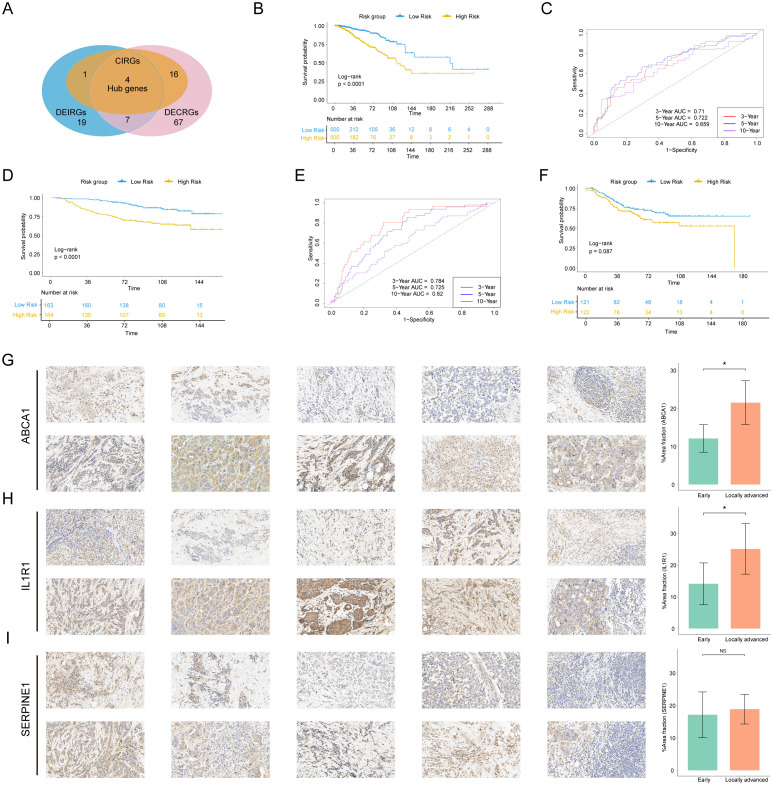
Construction and validation of the simplified 4-hub-gene prognostic model. **(A)** Venn diagram showing the overlap of 4 hub genes in DEIRGs, DECRGs and CIRGs. Kaplan-Meier survival curves of the 4-hub-gene prognostic model in the **(B)** TCGA cohort, **(D)** GSE20685 dataset, and **(F)** GSE21653 dataset. Time-dependent ROC curves for the prognostic model for 3-, 5-, and 10-year OS and the corresponding AUC values in the **(C)** TCGA cohort and **(E)** GSE20685 dataset. Representative IHC images and area fraction quantification for *ABCA1* **(G)**, *IL1R1* **(H)**, and *SERPINE1* **(I)** in early and locally advanced breast cancer tissues. *P < 0.05.

Within the CIRGs-based risk model, the 21 genes include those associated with coagulation responses as well as inflammatory responses. We performed Venn diagram analysis to intersect the CIRGs set with DECIRGs and DECRGs, yielding 4 hub genes functionally involved in both coagulation and inflammatory pathways (**Figure 5A**). Of these 4 hub genes, *ABCA1*, *IL1R1*, and *SERPINE1* were classified as CIRG-positive genes, whereas *HPN* was classified as a CIRG-negative gene. All three CIRG-positive hub genes exhibited significant positive pairwise correlations (*P* < 0.0001) (**Supplementary Figure 3A**). Furthermore, clinical correlation analysis revealed that the expression level of *IL1R1* exhibited statistically significant differences across different N stages and TNM stages (**Supplementary Figures 3B, C**), while *ABCA1* and *HPN* showed significant differences in expression across different M stages (**Supplementary Figures 3D, E**). Survival analysis confirmed that elevated expression of each hub genes was associated with poorer OS (**Supplementary Figures 3F–I**). Notably, patients with high expression of *HPN*—a CIRG-negative hub gene—had a better short-term prognosis but a poorer long-term prognosis. This finding indicates that *HPN* exerts different directions or magnitudes of impact on survival in different time periods.

To enhance the translational feasibility of the CIRGs-based model into clinical practice, we further developed a simplified prognostic model based solely on four core hub genes. This model integrates the expression levels of the four core hub genes with three independent prognostic clinical factors (age, T stage, and N stage). In the TCGA cohort, Kaplan-Meier curve analysis demonstrated that patients in the high-risk score group had significantly worse prognostic outcomes than those in the low-risk group (**Figure 5B**; *P* < 0.0001). Furthermore, ROC curves were employed to evaluate the predictive efficacy of the risk model. The AUC values for 3-year, 5-year, and 10-year OS were 0.71, 0.722, and 0.659, respectively (**Figure 5C**). We validated the predictive performance of the 4-hub-gene model using two independent external datasets. Kaplan-Meier curve analyses confirmed that breast cancer patients in the high-risk score group had an elevated risk of death (*P* < 0.001; *P* = 0.087) (**Figures 5D, 5F**). In the GSE20685 dataset, the 4-hub-gene model achieved AUC values of 0.784 and 0.725 for 3-year and 5-year OS, respectively (**Figure 5E**). In the GSE21653 dataset, the corresponding AUC value for 1-year OS was 0.731 (**Supplementary Figure 3J**). The 4-hub-gene model has lower detection costs and can significantly reduce the financial burden on patients.

To further validate the protein-level expression of three positively correlated CIRGs, namely *ABCA1*, *IL1R1*, and *SERPINE1*, we performed immunohistochemical (IHC) staining on breast cancer specimens across different disease stages. Quantitative analysis of the percentage of positively stained cell areas revealed that the expression levels of *ABCA1* and *IL1R1* were significantly higher in locally advanced breast cancer tissues than in early-stage samples (**Figures 5G, H**). By contrast, no statistically significant difference in *SERPINE1* expression was detected among specimens of different stages (**Figure 5I**), which is consistent with its low coefficient in the CIRGs-based model. These IHC findings underscore the potential involvement of *ABCA1* and *IL1R1* in breast cancer progression and highlight their potential as diagnostic and therapeutic targets.

### Validation of the involvement of CIRGs risk model based on scRNA-seq data

**Figure 6:**
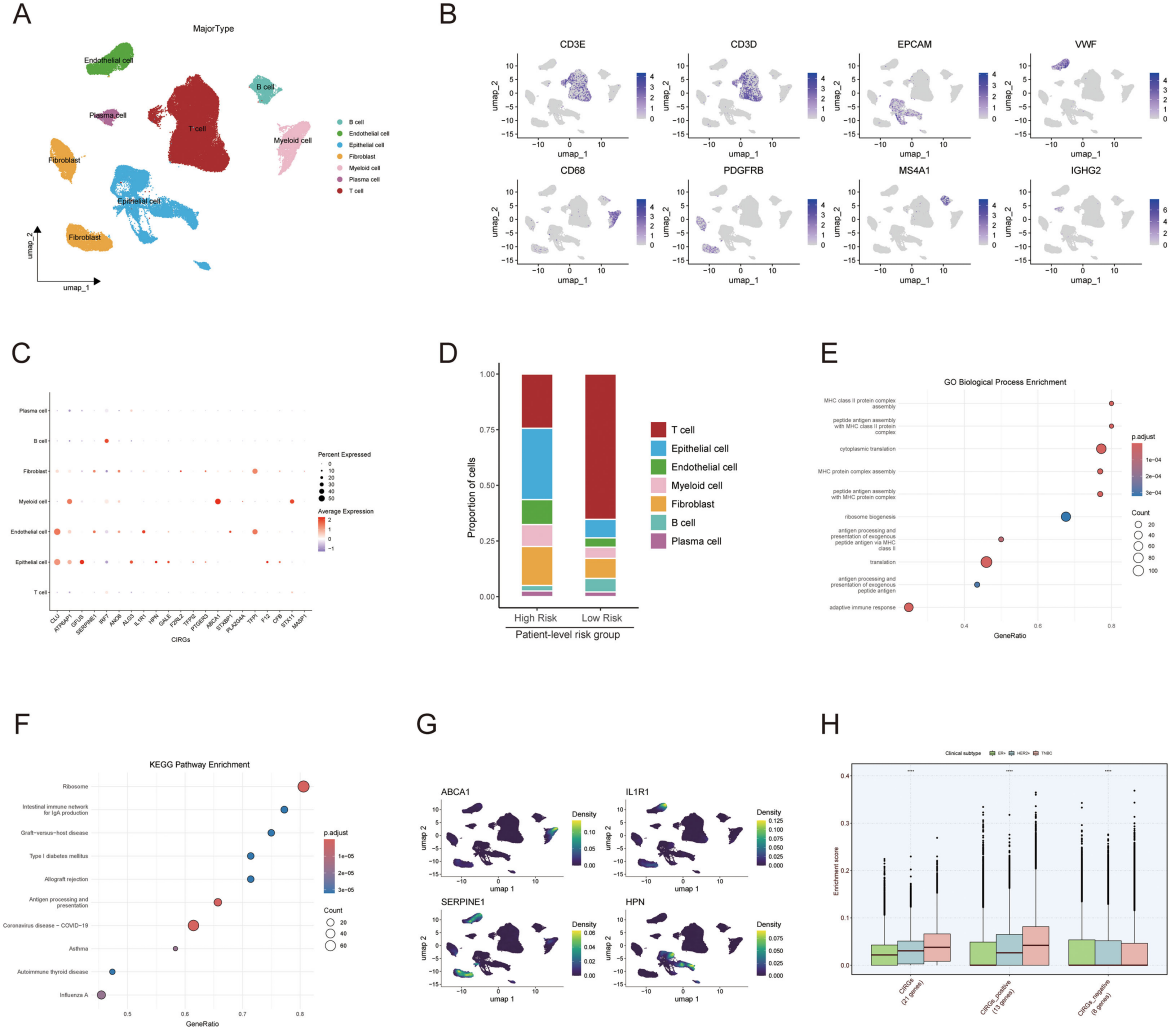
Single-cell transcriptomic profiling reveals cellular heterogeneity and molecular characteristics associated with CIRGs risk groups and clinical subtypes in breast cancer. **(A)** UMAP plot showing the distribution of major cell subsets. **(B)** UMAP showing the expression of marker genes for each cell type. **(C)** Bubble plot showing the expression of 21 CIRGs across seven cell types. **(D)** Stacked bar chart showing the proportional distribution of different cell types in high risk and low risk groups at the patient level. **(E)** Bubble plot showing the enrichment of GO biological processes for DEGs. **(F)** Bubble plot showing the enrichment of KEGG pathway for DEGs. **(G)** Gene expression density plot showing the distribution of four hub genes. **(H)** Box plot showing the distribution of enrichment scores of different clinical subtypes across the 3 CIRGs-related gene sets. Kruskal–Wallis tests was used to assess the significance of differences in panel **H**. ****P < 0.0001.

To further elaborate on the specific mechanisms by which the CIRGs-based model influences the TME of breast cancer, we analyzed the GSE176078 scRNA-seq dataset, which comprises 10 TNBC, 5 HER2-positive, and 11 ER-positive samples (23). We characterized cell types using marker genes, thereby identifying 7 major cell subpopulations: endothelial cells, B cells, epithelial cells, fibroblasts, myeloid cells, plasma cells, and T cells (**Figure 6A**). The UMAP visualization was applied to display the distribution of different cell types across various patients and clinical subtypes (**Supplementary Figures 4A, B**). **Figure 6B** showed the marker genes of the 7 cell subpopulations via UMAP. The top 10 DEGs in the cell subpopulations were presented in **Supplementary Figure 4C**. Importantly, the proportions of these major cell subpopulations varied across different clinical subtypes, with the results indicating a significant enrichment of immune cells in TNBC (**Supplementary Figure 4D**).

We first evaluated the expression profiles of the 21 genes composing the CIRGs model across the 7 major cell subpopulations and found that CIRG-related genes were predominantly expressed in myeloid cells, endothelial cells, and epithelial cells (**Figure 6C**). Subsequently, we scored each cell based on the CIRGs risk model. As expected, plasma cells, T cells, and B cells exhibited the lowest CIRGs risk scores, suggesting that the CIRGs may regulate immune infiltration primarily through myeloid cells, endothelial cells, and epithelial cells (**Supplementary Figure 4E**). Patients were categorized into distinct risk groups according to the CIRGs model, with the low-risk group exhibiting higher levels of immune infiltration and lower tumor burden (**Figure 6D**). The results of scRNA-seq analysis corroborated the robustness of our previous bulk sequencing findings (**Figure 4D**; **Supplementary Figure 2A**). To screen for genes with differential expression between high- and low-risk patients, comparisons between the two risk groups were performed for each cell cluster. The volcano plot illustrated the top 10 upregulated and downregulated genes from the DEGs analysis across 7 clusters (**Supplementary Figure 4F**). GO enrichment analysis of the differentially expressed genes revealed significant enrichment of signaling pathways related to antigen presentation, including the MHC-mediated signaling pathway and antigen processing and presentation of exogenous peptide antigen (**Figure 6E**). KEGG enrichment analysis of the DEGs also demonstrated enrichment of the antigen processing and presentation pathway (**Figure 6F**).

Given that we had previously identified four hub genes involved in both coagulation and inflammatory responses, we visualized the nuclear density of these four hub genes. The results showed that *ABCA1* was predominantly expressed in myeloid cells, *IL1R1* and *SERPINE1* were mainly expressed in endothelial cells and fibroblasts, while *HPN* was expressed in epithelial cells with relatively low expression levels in other cell subpopulations (**Figure 6G**). Gene co-expression analysis revealed that the four hub genes were significantly highly expressed in endothelial cells (**Supplementary Figure 4G**), which reflects the core role of endothelial cells in inflammatory and coagulation responses.

We calculated the scores of CIRGs-related gene sets using the AUCell and found that the CIRGs were significantly enriched in TNBC. Concurrently, the 13 genes positively correlated with the CIRGs risk model were also markedly enriched in TNBC. Notably, the 8 genes negatively correlated with the CIRGs risk model were enriched in ER+ breast cancer and lowly expressed in TNBC, with the enrichment scores of the CIRGs-negative correlation gene set being zero in most cells (**Figure 6H**). The enrichment of the CIRGs set, particularly the CIRGs-positive correlation set, in TNBC was consistent with the high expression of the clinical indicator SCI in TNBC (**Figure 1B**), further reflecting that the intensity of coagulation and inflammatory responses is closely correlated with molecular subtypes of breast cancer.

### Differential intercellular communication between patients in the CIRGs risk groups

**Figure 7:**
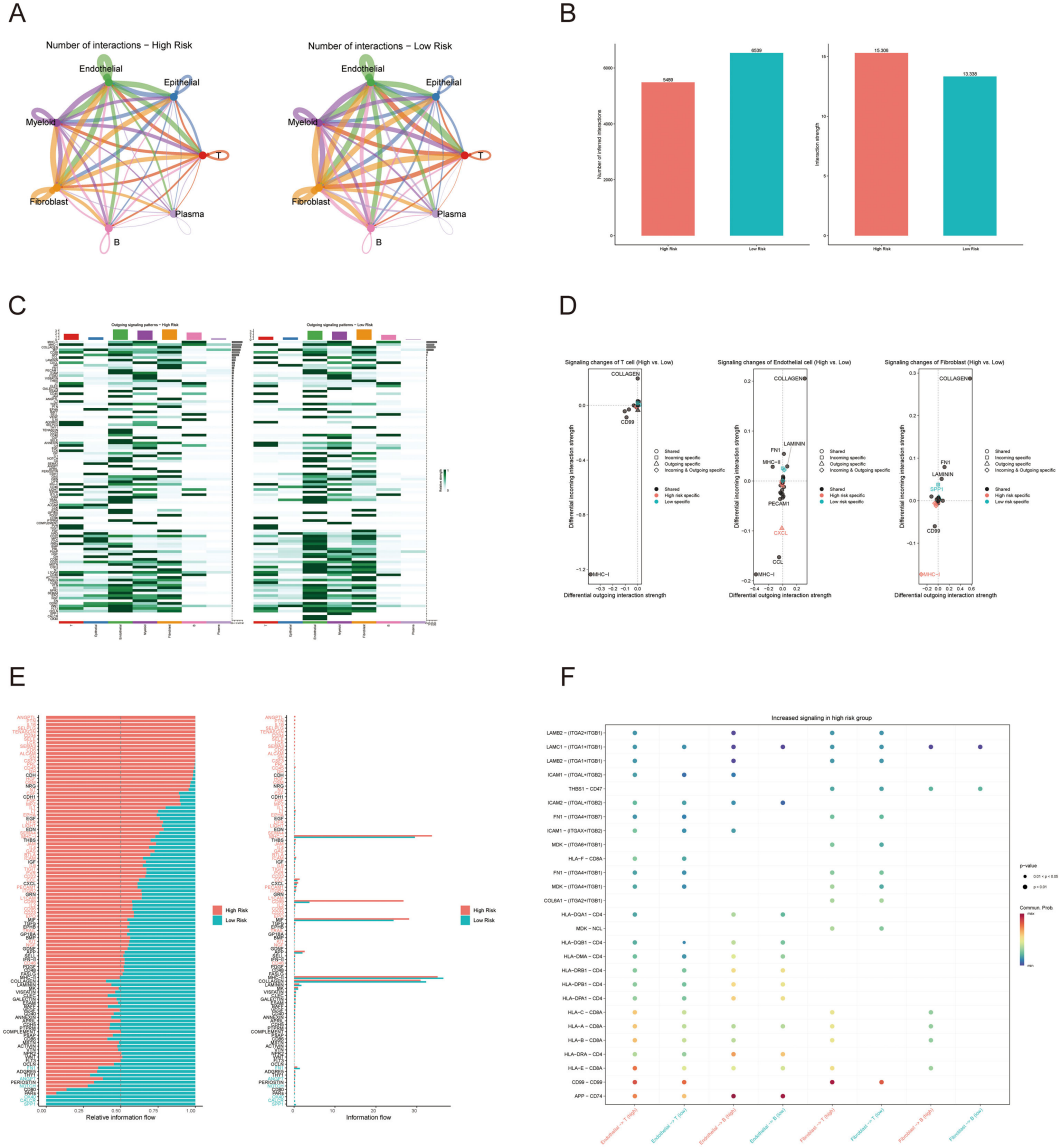
Comparison of cell-cell interactions between patients in the high- and low-risk groups. **(A)** Circle plots showing the number of interactions between different cell populations in patients from the high- (left) and low-risk (right) groups. **(B)** Bar graphs showing the number (left) and strength (right) of ligand-receptor interactions in patients from the high- and low-risk groups. **(C)** Heatmap showing the outgoing signal patterns associated with each cell population in patients from the high- and low-risk groups. **(D)** Identification of signal changes in T cells (left), endothelial cells (middle), and fibroblasts (right). **(E)** Comparison of information flow intensities between patients in the high- and low-risk groups. **(F)** Dot plot showing the upregulated ligand-receptor pairs in patients from the low-risk group.

To compare the cell communication patterns between the two CIRGs risk groups and explore potential therapeutic targets, we used gene expression data from single-cell transcriptomics as input and integrated interactions between ligands, receptors, and their cofactors to simulate intercellular communication. We observed that fibroblasts, myeloid, and endothelial cells in the high-risk group exhibited the highest number of interactions, indicating that these three cell types possess active biological functions (**Figure 7A**). **Figure 7B** illustrated that the high-risk group had fewer interactions compared to the low-risk group, while the signal strength in the high-risk group was higher. In the low-risk group, the number of signaling pathways from fibroblasts and endothelial cells to T/B cells was notably higher than that in the high-risk group. However, the strength of signaling pathways from myeloid cells and endothelial cells to T/B cells in the low-risk group was reduced (**Supplementary Figure 5A**). Furthermore, heatmaps showed that the outgoing signal flow of endothelial cells and the incoming signal flow of T cells were reduced in the low-risk group (**Figure 7C**; **Supplementary Figure 5B**). Specifically, in the low-risk group, collagen expression in T cells was increased, and the expressions of collagen, FN1, and laminin in endothelial cells and fibroblasts were elevated; in the high-risk group, MHC-I and CD99 expressions in T cells and fibroblasts were upregulated, while the expressions of MHC-I, CXCL, and CCL in endothelial cells were upregulated (**Figure 7D**).

In the high-risk group, multiple growth factor-associated proliferation pathways and cytokine-associated inflammation pathways participated in intercellular signal transduction mediated by ligand-receptor pairs, such as pathways related to FGF, IL6, TGF-β, and TRAIL. In contrast, intercellular interactions in the low-risk group were mainly active in MHCII, collagen, FN1, ANGPT, and SPP1 signaling pathways (**Figure 7E**). Given the high expression of hub genes in endothelial cells and fibroblasts as well as their key roles in intercellular communication, we also evaluated the frequency of ligand-receptor communication between endothelial cells/fibroblasts and T/B cell subsets in the two groups. Ligand-receptor pair analysis revealed that endothelial cells in the high-risk group preferentially transmitted signals to T cells and B cells via LAMB2-(ITGA1/2+ITGB1) (**Figure 7F**). Collectively, our findings emphasize that the complex intercellular signal networks in the CIRGs risk groups are orchestrated by ligand-receptor pairs, thereby leading to the TME differences. Targeting the high-risk group-specific cell-cell communication patterns may be a promising strategy for treatment.

## Discussion

Current prognostic assessment and personalized treatment strategies for breast cancer have limitations (34, 35), underscoring the need for further research focused on identifying novel, reliable prognostic biomarkers. Complex crosstalk between coagulation, inflammation, and malignancy plays a pivotal role in tumor initiation, progression, metastatic dissemination, and clinical prognosis, and has emerged as an increasingly prominent focus of cancer research (12, 13). In this study, we demonstrated that peripheral blood SCI can serve as a predictive survival indicator for breast cancer, providing a clinically valuable tool for clinicians to evaluate patient prognosis. Leveraging transcriptomic data from the GEO database and the TCGA-BRCA cohort, we developed a CIRG-based risk signature and elucidated the contributions of coagulation and inflammatory responses to the breast cancer tumor immune microenvironment, therapeutic response, and prognosis at the single-cell transcriptomic level.

SCI reflects the systemic coagulation-inflammation balance state of the whole body induced by the tumor. In contrast, the CIRGs risk signature reflects the molecular characteristics of coagulation-inflammation in the local tumor microenvironment. The biological information they carry is complementary rather than completely overlapping. SCI has the advantages of convenient detection, extremely low cost, and high reproducibility. It is suitable as a first-line preliminary prognostic stratification tool in clinical practice, especially in regions with limited medical resources, to quickly evaluate the systemic coagulation-inflammatory status of patients before treatment and assist in basic treatment decision-making. The CIRGs signature is a more precise molecular stratification tool. Beyond prognostic prediction, it can reflect the immune microenvironment status of the tumor and predict sensitivity to multiple therapeutic drugs. It is suitable for more refined molecular subtyping of patients to guide individualized selection of targeted therapy.

Coagulation and inflammation biomarkers in cancer patients can be easily obtained from routine blood tests and serve as auxiliary indicators for tumor prognosis assessment (36, 37). Currently, the main biomarkers that comprehensively reflect an individual’s coagulation and inflammation levels are PLR (16) and SCI (17). Hou et al. identified using machine learning that SCI is the most influential predictor of clinical deterioration in chronic obstructive pulmonary disease (18). PLR is a widely used inflammatory marker for tumor prognosis; previous researches have shown that elevated PLR is associated with pCR (38) and survival outcomes (39) in breast cancer. However, the prognostic value of SCI remains unvalidated in patients with cancer. In the present study, an SCI value of less than 106.5 was verified as an independent prognostic factor, and its predictive efficacy for 5-year and 8-year OS was superior to that of PLR in breast cancer. Notably, SCI levels tended to be elevated in TNBC. This finding aligns with prior studies, supporting the notion that peripheral blood biomarkers exert variable prognostic utility across breast cancer molecular subtypes (40).

According to the SCI calculation formula, elevated SCI may result from increased levels of platelets and fibrinogen or decreased leukocyte counts. This indicates that SCI reflects two interrelated yet distinct biological mechanisms: excessive activation of the coagulation pathway and systemic immunosuppression. Our study observed a strong positive correlation between SCI and the PLR, and elevated PLR is precisely the combined result of relative platelet increase and relative lymphocyte decrease, which directly confirms the synergistic contribution of these two mechanisms to elevated SCI levels. From the perspective of coagulation activation, tumor cells can promote hepatic fibrinogen synthesis (41), stimulate megakaryocyte proliferation and platelet production (42), and induce peripheral lymphocyte apoptosis and functional exhaustion (43) by secreting cytokines such as tissue factor and tumor necrosis factor-α. From the perspective of systemic immunosuppression, reduced leukocyte counts, particularly lymphocyte depletion, is a typical feature of advanced malignancies. Single-cell transcriptome analysis in this study revealed that the CIRGs high-risk group had significantly decreased numbers of tumor-infiltrating CD8^+^ T cells, B cells, and natural killer cells, which was highly consistent with the phenotype of peripheral blood lymphopenia. More importantly, these two mechanisms are closely interconnected: activated platelets can directly inhibit T cell function by releasing transforming TGF-β (44); the extracellular matrix barrier formed by fibrinogen deposition impedes lymphocyte infiltration into the tumor parenchyma (45).

Peripheral blood leukocytes are primarily composed of three major subsets: granulocytes, lymphocytes, and monocytes. Neutrophils account for approximately 60% of the total circulating leukocytes in humans and represent one of the most common immune cells in the TME (46). Tumor-associated neutrophils (TANs) exhibit two phenotypes: the anti-tumor N1 phenotype can directly kill tumor cells by secreting reactive oxygen species (47). They can also promote T cell activation and recruit pro-inflammatory macrophages, thereby shaping an immune-activated TME (48). Previous work has shown that targeting the activity of the arachidonic acid metabolism pathway can reduce the enrichment of N2-phenotype neutrophils in TNBC patients, thereby improving the response rate to immunotherapy (49). Lymphocytes are core components of the immune system and play critical roles in immune defense, immune surveillance, and immune regulation (50). Wu TD et al. reported that lymphocytes present in peripheral blood can be detected in tumor tissues (51); this finding suggests that a reduction in peripheral blood lymphocytes may decrease lymphocyte infiltration into tumor tissues. Tumor-infiltrating lymphocytes are aggregated in the tertiary lymphoid structures in the tumor invasive margin or stromal region. They have been proven to be effective prognostic biomarkers for various solid cancers (52). Monocytes serve as pivotal mediators of innate immune defense and tissue repair. Investigators from the Ludwig Institute for Cancer Research found that a relatively higher number of monocytes in tumors correlates with better prognosis in esophageal cancer patients receiving chemotherapy combined with immunotherapy (53).

Circulating platelet levels reflect both systemic coagulation and inflammatory status, and have been increasingly utilized to predict tumor prognosis in recent years (54). Platelets promote tumor growth and angiogenesis by secreting microvesicle secretion, and promote distant metastatic dissemination of cancer cells (55). As a key constituent of the coagulation cascade, fibrinogen can interact with integrins to increase tumor malignancy and promote tumor progression and metastasis (56). Furthermore, fibrinogen can also bind to vascular endothelial growth factor through vascular endothelial cadherin to promote angiogenesis (57). Therefore, fibrinogen is widely used to predict cancer survival outcomes and therapeutic response (58). Calculated from circulating leukocyte, platelet, and fibrinogen levels, SCI more comprehensively captures cancer-associated systemic coagulation and inflammatory status. To the best of our knowledge, this is the first study to investigate SCI as a prognostic biomarker in patients with cancer, and its prognostic utility across additional cancer types warrants further investigation.

Given the potential impact of CIRGs on breast cancer clinical outcomes, we constructed a CIRGs prognostic model based on 21 features identified by LASSO regression analysis. In the TCGA-BRCA cohort, higher CIRG signature scores were significantly associated with poorer OS. Survival analyses in the GSE20685 and GSE21653 external validation cohorts further confirmed that the CIRG-based risk signature maintained robust discriminative performance across independent patient cohorts. The three cohorts have significant differences in geographical distribution of enrolled populations, tumor stage composition, molecular subtype composition, and follow-up duration. Such real-world population heterogeneity is the most common reason for performance decline in the external validation of prognostic models, and can instead verify the universality of the model in different populations. Clinicopathological analysis revealed a higher proportion of patients with advanced-stage breast cancer in the high-risk group, which explains the lower survival rate in this group. We further integrated the CIRGs-based risk score with other independent prognostic factors to construct a nomogram, which exhibited improved predictive performance and higher net clinical benefit compared with the risk signature alone. We performed functional enrichment analysis to investigate the molecular mechanisms underlying the prognostic differences between the high- and low-risk groups. Enrichment results indicated that the low-risk group was notably enriched in pathways related to immune activation, such as immune receptor activity and cell killing pathways. Accordingly, we further explored the association between CIRGs scores and the tumor immune landscape. Our analyses showed that the infiltration levels of mast cells, natural killer cells, activated B cells, and activated CD8+ T cells were significantly higher in the low-risk group, whereas M2 macrophages were significantly enriched in the high-risk group. M2 macrophages drive tumor metastasis progression by suppressing anti-tumor T cell immunity and promoting angiogenesis, a phenotype strongly linked to unfavorable survival outcomes (31). Thus, compared with the high-risk group, patients were more prone to harbor “immune-hot” tumors in the low-risk group, which may lead to a better prognosis. Consistently, this finding was further validated using the scRNA-seq data employed in this study.

This study exclusively enrolled patients receiving standard NACT in the clinical cohort, whereas postoperative adjuvant treatment backgrounds were heterogeneous across the TCGA and GEO cohorts. Strict quality control was implemented during study design to address such discrepancies: all hematological parameters in the clinical cohort were collected within one week prior to NACT initiation, which completely eliminated the direct interference of chemotherapy on peripheral blood coagulation-inflammatory biomarkers. No significant correlation was observed between SCI and post-NACT pCR, further demonstrating that SCI reflects the baseline intrinsic biological characteristics of tumors rather than chemotherapy sensitivity, thereby conferring NACT-independent prognostic value. Unlike peripheral blood SCI, the CIRGs model was constructed based on transcriptomic data from primary tumor tissues, with nearly all breast cancer specimens collected from treatment-naive patients. The CIRGs model exhibited robust and stable predictive performance across multiple external validation cohorts, indicating that it captures conserved coagulation-inflammation-related biological processes in breast cancer with favorable generalizability.

ICB therapy has achieved substantial clinical advances in breast cancer across diverse molecular subtypes and disease stages (59, 60). Nevertheless, only a small proportion of breast cancer patients can derive benefits from immunotherapy. In our study, we found that CIRGs were associated with the anti-PD-1 treatment pathway, indicating that coagulation and inflammation play important roles in predicting immunotherapeutic responses. Specifically, the high-CIRGs subgroup exhibited a negative correlation with immune scores, suggesting that patients with reduced CIRGs levels are likely to achieve favorable responses to ICB therapy. Interestingly, the CIRG signature was correlated with the potential for immunotherapy response targeting immune checkpoints, although the underlying biological mechanisms require further investigation. Additionally, we observed that the IC50 values of drugs such as acetalax, mitoxantrone, niraparib, sabutoclax, PCI-34051, and topotecan were significantly correlated with the CIRGs prognostic signature score. As a classic topoisomerase II inhibitor, the association of mitoxantrone with coagulation-inflammation is not independent of its anticancer activity; rather, it shares identical molecular pathways with its multitargeted anticancer mechanisms, such as FAK kinase inhibition and HIF-1α translational inhibition (61). As a highly selective PARP inhibitor, niraparib directly regulates coagulation activation and chronic inflammation in the TME upstream by inhibiting the non-DNA repair functions of PARP-1 and reducing cytokine levels (62). The primary anticancer mechanism of topotecan is to induce DNA damage and apoptosis in tumor cells via inhibition of topoisomerase I. Meanwhile, topotecan also suppresses the activation of vascular endothelial cells and platelets through topoisomerase I inhibition, thereby blocking the critical initiating step of coagulation (63). Among these drugs, acetalax was the only one to which the low-risk group was sensitive. As a derivative of 5-aminosalicylic acid, acetalax may potentially activate the immune microenvironment by modulating the expression of inflammation-related genes and proteins, and exert synergistic effects with immunotherapy (64). However, the specific mechanisms of action of these drugs on the coagulation-inflammation pathway and their actual efficacy in patients with different risk stratifications remain to be further validated through *in vivo* animal experiments and prospective clinical trials. The above evidence demonstrates that our prognostic score provides an alternative strategy for the precise management of breast cancer patients and may hold great potential for guiding personalized therapy.

After screening based on CIRGs, we identified four hub genes (*ABCA1*, *IL1R1*, *SERPINE1*, and *HPN*) from the 21 genes, which are functionally involved in both coagulation and inflammatory pathways. Among these four hub genes, *HPN* was negatively correlated with the CIRG signature risk score and was identified as a protective factor for short-term breast cancer survival. *HPN* is a biomarker for distinguishing normal tissues from prostate cancer lesions (65). Mechanistic analysis showed that *HPN* is mainly associated with protease decomposition activity, and promotes cancer initiation and progression by affecting cell proliferation, epithelial-mesenchymal transition, inflammation, and tyrosine-kinase signaling pathways (66). Interestingly, Fang et al. demonstrated that low *HPN* expression is associated with increased susceptibility to hepatocellular carcinoma and poorer prognosis. Low *HPN* expression promotes extensive infiltration of tumor-associated macrophages, regulatory T cells, and other immunosuppressive cells (67). These previously reported findings may explain the time-dependent dual survival effect of *HPN*. Latest studies have indicated that *ABCA1* functions as a key regulator of cholesterol metabolism, mediating reverse cholesterol transport to reduce intracellular cholesterol levels (68). In papillary thyroid carcinoma, *ABCA1* is abnormally expressed in highly invasive cell lines, and its upregulation is associated with increased cell migration and invasion (69). Lappano et al. reported that *IL1R1* is associated with the invasive phenotype of both TNBC cells and cancer-associated fibroblasts (70). In colorectal cancer, fibroblast-specific knockout of *IL1R1* reduces intratumor Th17 cell infiltration and alleviates tumor growth *in vivo* (71). *SERPINE1* serves as a core regulator of the plasminogen/plasmin system and is well established as a prognostic biomarker in gastric cancer (72) and colorectal cancer (73).

Single-cell data analysis revealed that T/B cells exhibited lower CIRGs scores, and immune cells were higher in patients of the low-risk group. Differences in cell subsets were observed between different CIRGs prognostic subgroups and molecular subtypes, which may be associated with heterogeneity and microenvironment remodeling during tumor initiation and progression (74). We found that endothelial cells co-expressed all four hub genes. A growing body of evidence indicates that endothelial cells serve as a key barrier between blood and vessel walls and precisely regulate the balance between coagulation and anticoagulation (75). Similarly, endothelial cells control the recruitment and egress of immune cells during inflammatory processes (76). Interestingly, as reflections of the same pathological process at different dimensions, both the gene-level marker (CIRGs risk model) and the blood marker (SCI) were higher in TNBC patients, suggesting a strong correlation between the two biomarkers. This implies that local genes may drive systemic phenotypic changes. In the high-CIRGs group, we observed an overall increase in the intensity of intercellular communication, with active signaling pathways including FGF, IL6, and TGF-β. Aberrant activation of the FGF/FGFR pathway maintains cancer stem cell properties (77), which can lead to tumor recurrence and treatment resistance. The key downstream signaling pathway altered by IL-6 activation is the JAK/STAT3 pathway, which promotes continuous proliferation of tumor cells and inhibits their apoptosis (78). TGF-β can create an aggressive TME by stimulating peritumoral angiogenesis and suppressing immune responses (79). In the high-CIRGs group, a ligand-receptor pair, LAMB2-(ITGA1/2+ITGB1), involved in T cell adhesion and migration, was significantly increased. Previous studies have shown that LAMB2 is downregulated during anti-PD-1 therapy, can predict patient survival, and represents a promising potential drug target (80). These findings further confirm that in-depth investigation of changes in cell-cell interactions and downstream signaling pathways is crucial for deciphering tumorigenic mechanisms and identifying potential therapeutic targets.

Our studies consistently demonstrate that the systemic hematological biomarker SCI and tissue-based CIRGs signature are significantly elevated in TNBC, reflecting the most robust coagulation-inflammatory response among all breast cancer subtypes. TNBC is characterized by high genomic instability (e.g., TP53 mutations, BRCA1/2 deficiencies) and rapid cell proliferation (81), leading to extensive tumor cell necrosis and the release of proinflammatory cytokines (82). Upon entering the systemic circulation, these cytokines trigger systemic coagulation and inflammatory cascades. Meanwhile, systemic inflammation drives the recruitment and polarization of immunosuppressive cells into tumor tissue, further constructing an immunosuppressive TME (83). This is highly consistent with our finding that high CIRGs risk is associated with reduced infiltration of activated CD8 positive T cells and increased M2 macrophage infiltration in TNBC. Emerging clinical evidence indicates that in metastatic melanoma, anticoagulant therapy with factor Xa inhibitors has been shown to enhance the efficacy of ICB treatment by reversing fibrin-mediated immunosuppression (14). A systematic review has demonstrated that heparin significantly improves overall survival in patients with metastatic cancer through multiple mechanisms (84). The SCI and CIRGs models can identify TNBC patients with excessive activation of the coagulation-inflammation pathway, who are most likely to benefit from adjuvant anticoagulant therapy. However, it should be noted that the optimal combination regimen, dosage, and treatment duration remain to be determined. Large-scale, multicenter prospective clinical trials are urgently needed to validate the safety and efficacy of combined anticoagulant and ICB therapy in TNBC.

This study has several limitations. First, this work is fundamentally a retrospective observational study based on single-center clinical hematological data and public transcriptomic datasets. The retrospective study design is susceptible to inherent selection bias, and public transcriptomic datasets have unavoidable batch effects and detection platform differences, which may affect the generalizability of the model. The number of cases in our institutional SCI cohort is limited, and a large-scale prospective cohort is required to further validate the prognostic value of SCI. Second, although we analyzed the prognostic value and clinical significance of CIRGs and identified interactions between the immune microenvironment, coagulation, and inflammation using single-cell transcriptome data, the underlying biological mechanisms remain unclear. Although we have preliminarily verified the protein expression of several hub genes in a small sample of clinical tissues via immunohistochemistry, further functional and mechanistic experiments are needed to elucidate the roles of CIRGs, especially the four hub genes in breast cancer. Another limitation is the lack of individual-level paired validation between SCI and the CIRGs model within the same cohort. Due to the limitations of public databases and historical samples at our institution, we were unable to directly quantitatively analyze the correlation between CIRGs expression in tumor tissues and peripheral blood SCI levels in individual patients. Future prospective multicenter clinical studies are warranted to simultaneously collect blood samples and paired tumor tissue samples from patients, conduct systematic correlation analyses and validate whether the combined use of these two biomarkers can further improve the accuracy and clinical utility of breast cancer prognosis prediction.

## Conclusions

In this study, we are the first to confirm that SCI has stable prognostic predictive capacity for breast cancer patients, outperforming the predictive value of PLR. We developed a CIRGs-based risk model and identified four hub genes, and further constructed a nomogram to predict OS probability in breast cancer patients. The established prognostic model, combined with systematic analyses of the tumor immune landscape and cell-cell communication, provides valuable biomarkers for clinical decision-making.

## Data Availability

The original contributions presented in the study are included in the article/**Supplementary Material**. Further inquiries can be directed to the corresponding author.

## Supplementary material

Supplementary Table 1 Coagulation-related genes in this study.

Supplementary Table 2 Inflammation-related genes in this study.

Supplementary Table 3 Univariate cox regression survival analyses of the SCI for the prediction of DFS and OS.

Supplementary Table 4 Univariate and multivariate analysis of clinical characteristics and SCI in relation to pCR.

Supplementary Table 5 LASSO regression coefficients of 21 CIRGs.

Supplementary Figure 1 Integrate clinical risk factors and the risk model to predict the survival of breast cancer patients. **(A)** Heatmap showing the distribution of risk group and clinical risk factors. **(B)** Box plot showing the distribution of risk scores across different tumor stages. Nomograms of predicting survival probability for 3, 5, and 10 years in the **(C)** TCGA cohort, **(F)** GSE20685 dataset, and **(I)** GSE21653 dataset. Calibration plots for the 3-, 5-, and 10-year OS nomogram models in the **(D)** TCGA cohort, **(G)** GSE20685 dataset, and **(J)** GSE21653 dataset. Decision curve analysis for the clinical risk factors, risk group and Nomogram models built to predict 5-year OS probability in the **(E)** TCGA cohort, **(H)** GSE20685 dataset, and **(K)** GSE21653 dataset. Kruskal–Wallis tests was used to assess the significance of differences in panel **(B)**. ****P* < 0.001, **P* < 0.05.

Supplementary Figure 2 Immune landscape and drug sensitivity analysis of different risk groups. **(A)** Ridge plot showing the top 10 ranked pathways from the GSEA. **(B)** Box plots showing the infiltration differences of 22 immune cells between high and low risk groups. ****P* < 0.001, ***P* < 0.01, **P* < 0.05.

Supplementary Figure 3 Identification of hub genes and validation of their correlations with clinical indicators and prognosis. **(A)** Scatter plot showing the correlations between the expression levels of three hub genes (*ABCA1*, *IL1R1*, and *SERPINE1*) that are positively correlated with the risk model. **(B)** Box plot showing the distribution of *IL1R1* expression across different N stages. **(C)** Box plot showing the distribution of *IL1R1* expression across different tumor stages. **(D)** Box plot showing the distribution of *ABCA1* expression across different M stages. **(E)** Box plot showing the distribution of *HPN* expression across different M stages. **(F–I)** Kaplan-Meier survival curves of the 4 hub genes in the TCGA database. **(J)** Time-dependent ROC curves for the risk model for 1-, 3-, and 5-year OS and the corresponding AUC values in the GSE21653 dataset. Kruskal–Wallis tests was used to assess the significance of differences in panels **(B–E)**.

Supplementary Figure 4 Single-cell transcriptomic profiling reveals cellular heterogeneity and molecular characteristics associated with CIRGs risk groups and clinical subtypes in breast cancer. **(A)** UMAP plot showing high-quality cells annotated by patient origin (patients, n = 26). **(B)** UMAP plot showing high-quality cells annotated by cell cycles. **(C)** Heatmap showing the expression of the top 10 DEGs for each cell type. **(D)** Stacked bar chart showing the proportional distribution of different clinical subtypes across various cell types. **(E)** UMAP plot showing the distribution of cells into high risk and low risk groups at the cell level. **(F)** Volcano plot showing DEGs of each cell type between the high and low risk group. **(G)** Gene expression density plot showing the distribution of co-expression patterns of the four hub genes.

Supplementary Figure 5 Comparison of cell-cell interactions between patients in the high- and low-risk groups. **(A)** Circle plots comparing the number (left) and strength (right) of interactions between different cell populations in patients from the high- and low-risk groups. **(B)** Heatmap showing the incoming signal patterns associated with each cell population in patients from the high- and low-risk groups.
