# Supplementary material for: Establishment and evaluation of novel prognostic biomarkers based on systemic coagulation-inflammation index and related genes in breast cancer
Source: Front Immunol. 2026 Jul 22;17:1836706. doi: 10.3389/fimmu.2026.1836706 (PMC13437700; doi:10.3389/fimmu.2026.1836706)
Supplement: Supplementary file 1 [file Table3.docx]

Table S3

Univariate cox regression survival analyses of the SCI for the prediction of DFS and OS.

| Variable |  | DFS | |  | OS | |
| --- | --- | --- | --- | --- | --- | --- |
|  |  | Hazard ratio (95% CI) | *P* value |  | Hazard ratio (95% CI) | *P* value |
| **Age** | <40 | Ref. |  |  | Ref. |  |
|  | 40 to 60 | 1.11 (0.85-1.46) | 0.433 |  | 1.30 (0.92-1.83) | 0.141 |
|  | ≥60 | 1.00 (0.65-1.54) | 0.999 |  | 1.21 (0.71-2.07) | 0.484 |
| **BMI** | <25 | Ref. |  |  | Ref. |  |
|  | 25 to 30 | 1.19 (0.92-1.54) | 0.196 |  | 1.25 (0.91-1.73) | 0.170 |
|  | ≥30 | 1.44 (0.87-2.37) | 0.153 |  | 1.60 (0.88-2.91) | 0.126 |
| **cT** | T1+T2 | Ref. |  |  | Ref. |  |
|  | T3+T4 | 1.47 (1.11-1.94) | **0.007** |  | 1.93 (1.40-2.68) | **<0.001** |
| **cN** | N0 | Ref. |  |  | Ref. |  |
|  | N1-3 | 1.91 (1.17-3.13) | **0.010** |  | 3.03 (1.42-6.45) | **0.004** |
| **HR** | Negative | Ref. |  |  | Ref. |  |
|  | Positive | 1.00 (0.77-1.30) | 0.997 |  | 0.74 (0.55-1.01) | 0.061 |
| **HER2** | Negative | Ref. |  |  | Ref. |  |
|  | Positive | 1.22 (0.95-1.58) | 0.125 |  | 1.33 (0.97-1.81) | 0.076 |
| **Ki67** | ≤14% | Ref. |  |  | Ref. |  |
|  | >14% | 1.08 (0.84-1.40) | 0.547 |  | 1.24 (0.90-1.72) | 0.195 |
| **P53** | Negative | Ref. |  |  | Ref. |  |
|  | Positive | 0.93 (0.72-1.19) | 0.551 |  | 0.99 (0.73-1.34) | 0.936 |
| **Surgery** | Mastectomy | Ref. |  |  | Ref. |  |
|  | Conservative surgery | 0.79 (0.48-1.31) | 0.368 |  | 0.38 (0.16-0.93) | **0.034** |
| **pCR** | No | Ref. |  |  | Ref. |  |
|  | Yes | 0.65 (0.47-0.90) | **0.009** |  | 0.57 (0.38-0.87) | **0.009** |
| **SCI** | Low | Ref. |  |  | Ref. |  |
|  | High | 1.31 (1.03-1.67) | **0.030** |  | 1.53 (1.13-2.07) | **0.006** |
