# Supplementary material for: Establishment and evaluation of novel prognostic biomarkers based on systemic coagulation-inflammation index and related genes in breast cancer
Source: Front Immunol. 2026 Jul 22;17:1836706. doi: 10.3389/fimmu.2026.1836706 (PMC13437700; doi:10.3389/fimmu.2026.1836706)
Supplement: Supplementary file 2 [file Table4.docx]

Table S4

Univariate and multivariate analysis of clinical characteristics and SCI in relation to pCR.

| Variable | pCR(n=162) | Univariate analysis Odds ratio(95% CI) | *P* value |  | Multivariate analysis  Odds ratio(95% CI) | *P* value |
| --- | --- | --- | --- | --- | --- | --- |
| **Age** |  |  |  |  |  |  |
| <40 | 62(38.3%) | Ref. |  |  | Ref. |  |
| 40 to 60 | 82(50.6%) | 0.66(0.45-0.96) | 0.029 |  | 0.69(0.46-1.03) | 0.067 |
| ≥60 | 18(11.1%) | 0.80(0.44-1.46) | 0.470 |  | 0.96(0.50-1.82) | 0.894 |
| **BMI** |  |  |  |  |  |  |
| <25 | 100(61.7%) | Ref. |  |  |  |  |
| 25 to 30 | 58(35.8%) | 1.29(0.89-1.86) | 0.184 |  |  |  |
| ≥30 | 4(2.5%) | 0.41(0.14-1.18) | 0.097 |  |  |  |
| **cT stage** |  |  |  |  |  |  |
| T1+T2 | 142(87.7%) | Ref. |  |  | Ref. |  |
| T3+T4 | 20(12.3%) | 0.50(0.30-0.83) | **0.007** |  | 0.40(0.23-0.69) | **0.001** |
| **cN stage** |  |  |  |  |  |  |
| N0 | 20(12.3%) | Ref. |  |  |  |  |
| N1-3 | 142(87.7%) | 0.78(0.45-1.34) | 0.364 |  |  |  |
| **HR** |  |  |  |  |  |  |
| Negative | 93(57.4%) | Ref. |  |  | Ref. |  |
| Positive | 69(42.6%) | 0.25(0.18-0.37) | **<0.001** |  | 0.33(0.22-0.48) | **<0. 001** |
| **HER-2** |  |  |  |  |  |  |
| Negative | 79(48.8%) | Ref. |  |  | Ref. |  |
| Positive | 83(51.2%) | 3.23(2.25-4.64) | **<0.001** |  | 2.12(1.43-3.14) | **<0.001** |
| **Ki-67** |  |  |  |  |  |  |
| ≤14% | 28(17.3%) | Ref. |  |  | Ref. |  |
| >14% | 134(82.7%) | 2.71(1.74-4.20) | **<0.001** |  | 1.98(1.24-3.17) | **0.004** |
| **P53** |  |  |  |  |  |  |
| Negative | 96(59.3%) | Ref. |  |  |  |  |
| Positive | 66(40.7%) | 1.01(0.71-1.44) | 0.964 |  |  |  |
| **Surgery** |  |  |  |  |  |  |
| Mastectomy | 148(91.4%) | Ref. |  |  |  |  |
| Conservative surgery | 14(8.6%) | 1.33(0.70-2.51) | 0.381 |  |  |  |
| **PLR** |  |  |  |  |  |  |
| Low | 87(53.7%) | Ref. |  |  |  |  |
| High | 75(46.3%) | 0.83(0.59-1.18) | 0.296 |  |  |  |
| **SCI** |  |  |  |  |  |  |
| Low | 85(52.5%) | Ref. |  |  |  |  |
| High | 77(47.5%) | 0.88(0.62-1.25) | 0.490 |  |  |  |
